# Interaction of host immunity with HER2-targeted treatment and tumor heterogeneity in HER2-positive breast cancer

**DOI:** 10.1186/s40425-019-0548-6

**Published:** 2019-03-29

**Authors:** Gaia Griguolo, Tomás Pascual, Maria Vittoria Dieci, Valentina Guarneri, Aleix Prat

**Affiliations:** 1 Translational Genomics and Targeted Therapeutics in Solid Tumors, IDIBAPS, Barcelona, Spain; 2 Department of Medical Oncology, Hospital Clínic, Barcelona, Spain; 3 Department of Surgery, Oncology and Gastroenterology, University of Padova, Padova, Italy; 4 Medical Oncology 2, Istituto Oncologico Veneto IRCCS, Padova, Italy

**Keywords:** Breast cancer, HER2, Targeted treatment, Immunity, Tumor infiltrating lymphocytes, Immune checkpoints

## Abstract

Growing evidence suggests a clear role of the host immune system in HER2+ breast cancer. In addition, HER2+ breast cancer is generally considered more immunogenic than hormone receptor-positive (HR+)/HER2-, and specific molecular HER2+ subgroups (e.g. HER2-enriched disease) are more immunogenic than others (e.g. Luminal A or B). From a clinical perspective, the immune system plays a relevant prognostic role in HER2+ breast cancer and contributes to the therapeutic effects of trastuzumab. However, as more HER2-targeted agents become available, a better understanding of the role played by the immune system in modulating therapy response to different agents will be needed. Furthermore, the recent introduction in oncology of immune checkpoint inhibitors capable of unleashing anti-tumor immune response opens new possibilities for therapeutic combinations in HER2+ breast cancer. Here, we review the current pre-clinical and clinical data on the interplay between the immune system and HER2+ breast cancer, focusing on different HER2-targeted treatments and the biological heterogeneity that exists within HER2+ disease. Finally, we discuss new therapeutic approaches exploiting the immune system to increase activity or revert resistance to HER2-targeted agents.

**Electronic supplementary material:**

The online version of this article (10.1186/s40425-019-0548-6) contains supplementary material, which is available to authorized users.

## Introduction

HER2 is overexpressed in 15–20% of breast cancers (BC) and is associated with clinically aggressive disease [1]. Targeting this oncogene has led to striking improvements in survival outcomes for HER2+ BC patients. To date, several HER2-targeted treatments are available, including monoclonal antibodies (trastuzumab, pertuzumab), tyrosine kinase inhibitors (lapatinib, neratinib), and antibody–drug conjugates (Ado-trastuzumab emtansine [T-DM1]) [2–6].

The role of the host immune system in HER2+ BC is becoming an important topic to study for several reasons. First, HER2+ BCs have higher stromal tumor-infiltrating lymphocytes (TILs) levels in general than hormone receptor positive (HR+)/HER2- BCs, implying that HER2+ disease is usually more immunogenic [7, 8]. Second, not all HER2+ tumors are immunogenic and specific molecular HER2+ subgroups (e.g. HER2-enriched) are more immunogenic than others (e.g. Luminal A/B) [9]. Third, the percentage of TILs is clinically relevant due to its association with better prognosis [10, 11]. Fourth, the recent introduction in oncology of therapeutic agents capable of unleashing anti-tumor immune response, such as checkpoint inhibitors, opens new treatment strategies [12]. Finally, the immune system not only plays a prognostic role but also seems to contribute substantially to the therapeutic effects of trastuzumab, originally credited to induce cell death by direct inhibition of HER2 intracellular signaling [13].

To date, several reviews have analyzed the prognostic role of immunity in HER2+ BC and its capability of modulating response to trastuzumab [13]. However, as more HER2-targeted agents have become available in recent years, better understanding of the role played by the immune system in modulating response to these new treatments might help optimize or tailor treatment. Furthermore, a deeper understanding of the interaction between immunity and combination of anti-HER2 drugs with hormonotherapy and chemotherapy, in the context of the biological heterogeneity within HER2+ BC, will be required to design biologically meaningful therapy combinations.

### HER2 as the antigen

HER2 overexpression in BC is often described as a typical case of oncogene addiction, thus defining tumors that are almost exclusively dependent on a single oncogenic pathway. As is the case for many oncogenes, its overexpression on the cell membrane and its essential role in tumor cell biology makes it a perfect antigen to guide immune response towards HER2+ cells [14].

In the attempt to induce host immune response towards HER2 for therapeutic uses, vaccines have been designed. A number of HER2-derived peptides have been investigated [15] and some have been shown capable of inducing immune response. However, despite some positive results in phase I-II trials, the development of BC vaccines has been a story of setbacks and efficacy has not been proved in phase III trials (Additional file 1: Table S1) [16].

**Fig. 1:**
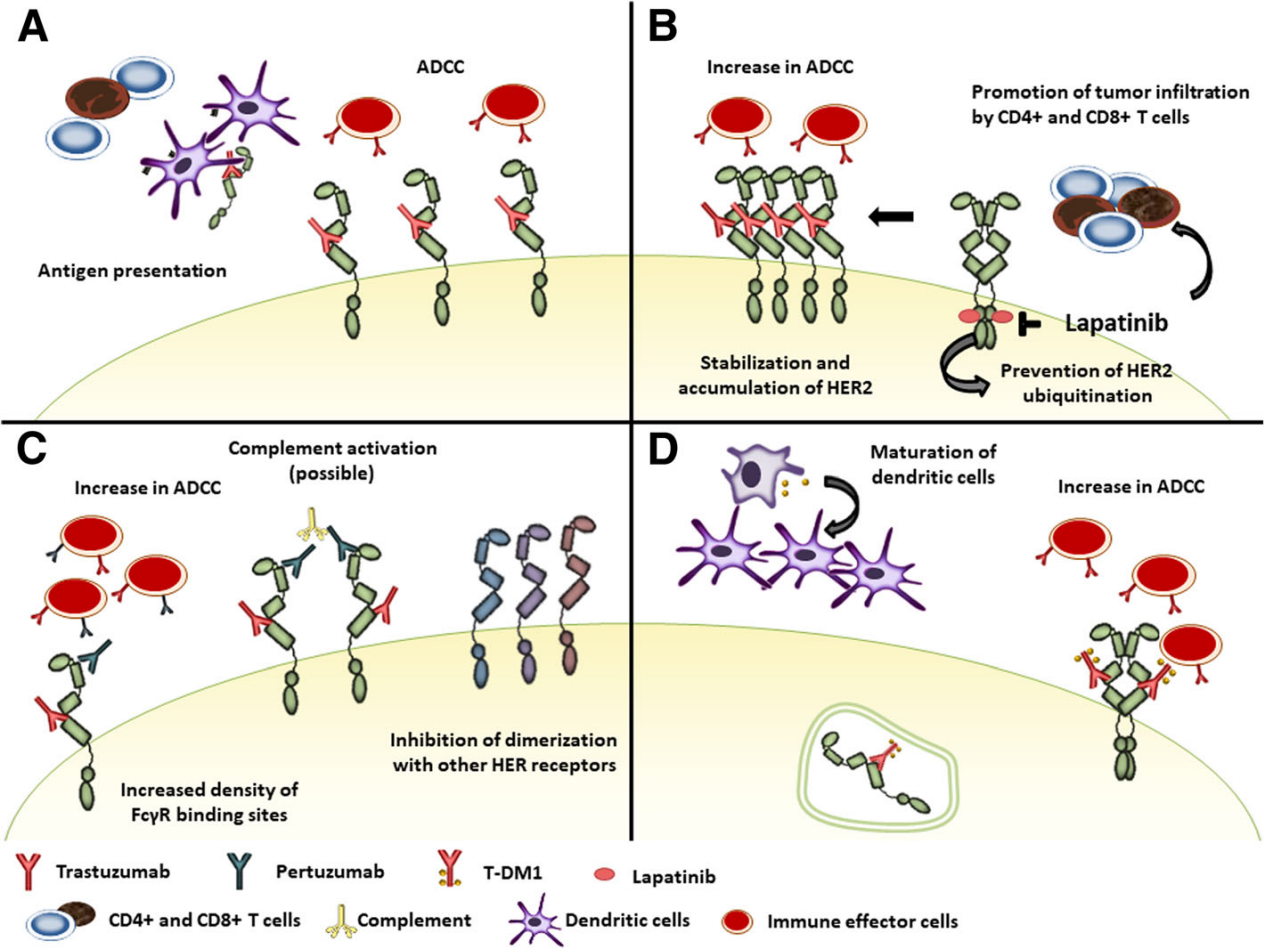
Immune related mechanisms of action of HER2-targeted agents: trastuzumab (**a**), lapatinib (**b**), pertuzumab (**c**), T-DM1 (**d**)

On the other hand, host immune response plays a key role in the activity of anti-HER2 monoclonal antibodies. Indeed, trastuzumab has several mechanisms of action (Fig. 1a). By binding to the extracellular domain of HER2, it prevents receptor dimerization inhibiting downstream signaling. It also increases HER2 internalization and endocytic degradation, thus enhancing HER2 peptide presentation on major histocompatibility complex (MHC) receptors. In addition, while the antibody binds to HER2 on the cell surface, the crystalline fragment (Fc) of the immunoglobulin interacts with Fc-gamma-receptors (FcγR) on innate immune effector cells, like natural killer (NK) cells, neutrophils and γδT-cells, activating antibody-dependent cellular cytotoxicity (ADCC) [17, 18]. This cytolytic activity increases availability of tumor antigens in the tumor microenvironment (TME), favoring antigen presentation. Antigen presentation is also enhanced by FcγR-mediated phagocytosis of immune complexes by antigen-presenting cells. Hence, the interaction between trastuzumab and the innate immune system facilitates the development of tumor-specific T-cell immunity. On one hand, NK-cells prime dendritic cells, enhancing tumor antigen presentation to cytotoxic CD8+ T-cells and polarization of CD4+ T-cells towards an anti-tumor T-helper type 1 (Th1) phenotype. On the other hand, trastuzumab-dependent NK-cell activation leads to cytokine secretion contributing to the recruitment and functional polarization of myeloid and T-cells [19].

Through these mechanisms, anti-HER2 antibodies exert a vaccine-like effect activating the adaptive as well as the innate immune system. Consistently, activation of anti-HER2 CD4+ Th1 response correlates with pathologic complete response (pCR) and disease-free survival (DFS) following anti-HER2 based neoadjuvant chemotherapy [20, 21].

### Evaluating immunity in BC

Generally, TILs are typically believed to reflect immunological response; however, they include different cell types usually dominated by T-cells, with variable proportions of B-cells, NK cells, macrophages and dendritic cells. While CD8+, CD4+ Th1 and NK cells are generally considered to favor a tumor-suppressive response, CD4+ T-helper 2 (Th2), FOXP3+ T-regulatory and dendritic cells might play a pro-tumorigenic role [22].

TILs are easily assessed on hematoxylin/eosin stained slides, both in intratumoral and stromal areas (sTILs). Current recommendation is to use sTILs as principal parameter.

However, all mononuclear cells are scored and semi-quantitative evaluation of TILs does not distinguish specific immune cells subtypes. Moreover, no clear cutoff exists to define what is a high infiltrate. Traditionally, the lymphocyte predominant BC (LPBC) definition (≥ 50–60% sTILs) has been used. More recently, TILs are measured as a continuous parameter to better represent the continuity of immune response [22, 23].

Gene-expression analysis can also be used to infer proportions of infiltrating immune cell populations, providing more information regarding different lymphocyte subpopulations, and to measure immune checkpoint gene expression [24]. Interestingly, checkpoint expression significantly correlates with other immune markers and TILs [25, 26].

### Host immunity in HER2 + BC treated with chemotherapy and trastuzumab

#### Prognostic role of baseline immunity in early HER2+ BC

To date, most data regarding the clinical validity of pre-existing immune response in HER2+ BC come from patients treated with trastuzumab-based chemotherapy for early BC (Additional file 1: Table S1 and Table S2). From a prognostic perspective, several studies in HER2+ BC patients receiving neoadjuvant (Table 1) or adjuvant [7, 24, 27–30] anti-HER2-based chemotherapy have shown that expression of immune-associated gene signatures and infiltration by TILs in pre-treatment biopsies associated with longer DFS [10], independently of known prognostic clinical-pathological variables.

**Table 1:** Neoadjuvant trials with trastuzumab-containing regimens which assessed the prognostic values of TILs and immune related gene signatures

Study	Treatment	N. pts.^a^	Biomarker Tested	Outcome Tested	Association
CALGB 40601 [100] NCT00770809	P-H P-L P-HL	265	Immune gene signatures [100]	pCR	IgG signature independently associated with pCR at multivariate analysis
CherLOB [55] NCT00429299	P-H → FEC-H P-L → FEC-L P-HL → FEC-HL	105	TILs [55]	pCR	Associated with pCR at univariate analyses (no statistical significance beyond PAM50)
				EFS	Associated with EFS at univariate analyses
		86	Immune gene signatures [55]	pCR	3 out of 4 signatures maintained association with pCR after correction for PAM50
GeparQuattro [57] NCT00288002	EC-H → D-H +/−X	178	TILs [57]	pCR	Associated with pCR at multivariate analysis
GeparQuinto [57] NCT00567554	EC-H → D-H	162	TILs [57]	pCR	
	EC-L → D-L	158		pCR	Not associated with pCR
GeparSixto [38] NCT01426880	PM-HL +/− C	266	TILs [38]	pCR	Associated with pCR at multivariate analyses
		226	mRNA expression of immunologic genes	pCR	All 12 immune mRNA markers were associated with pCR (10/12 at multivariate analysis)
NeoALTTO [58] NCT00553358	P-H P-L P-HL	387	TILs [58]	pCR	Associated with pCR at multivariate analysis
				EFS	Associated with EFS at multivariate analysis
		254	Immune gene signatures [59]	pCR	two T-cell immune signatures were associated with pCR (only confirmed at multivariate analysis in P-HL arm)
NOAH [75] ISRCTN86043495	AP → P → CMF	51	Four immune metagenes [45]	pCR	Not associated with pCR
	AP-H → P-H → CMF-H → H	63		pCR	3/4 associated with pCR
NeoSphere [45] NCT00545688	DH DPrtz DHPrtz HPrtz	243	TILs [45]	pCR	Not significantly associated with pCR
		305	PDL1 by IHC [45]	pCR	Not significantly associated with pCR
		337	Immune genes and metagenes [45]	pCR	5 associated with pCR at multivariate analysis (different results in the DHPrtz arm)
Tryphaena [44] NCT00976989	FEC → DHPrtz FECHPrtz→DHPrtz CycloDHPrtz	213	TILs [43]	pCR	Not significantly associated with pCR
				EFS	Associated with EFS at multivariate analysis
		173	Immune signatures and genes [43]	pCR	2 signatures and 4 genes associated with pCR at multivariate analysis
				EFS	Not associated with EFS at multivariate analysis
PAMELA [9] NCT01973660	HL	134	TILs at day15	pCR	Associated with pCR at multivariate analysis

^a^ Number of patients included in the biomarker analysis

*A* doxorubicin, *C* carboplatin, *Cyclo* cyclophosphamide, *CMF* cyclophosphamide-methotrexate-fluorouracil, *D* docetaxel, *EC* epirubicin-cyclophosphamide, *EFS* event-free survival, *FEC* fluorouracil-epirubicin-cyclophosphamide, *H* trastuzumab, *IHC* immunohistochemistry, *L* lapatinib, *P* paclitaxel, *pCR* pathologic complete response, *PM* weekly paclitaxel + non pegylated liposomal doxorubicin, *Prtz* pertuzumab, *TIL* tumor infiltrating lymphocytes, *X* capecitabine

Data from the GeparQuattro trial and from the EC-H→D-H arm of the GeparQuinto trial were analyzed jointly

#### Role of immunity in residual disease after neoadjuvant treatment

Timing of TILs evaluation might be important. In residual disease after neoadjuvant therapy, TILs might have a different prognostic meaning. In a retrospective study, including 175 HER2+ BC patients treated with neoadjuvant chemotherapy+/−trastuzumab, sTILs generally decreased during treatment (78% of patients). Presence of high TILs (> 25%) in patients with residual disease after neoadjuvant therapy was associated with worse DFS [31]. This pattern is opposite to that reported for triple-negative BC (TNBC), where high TILs in residual disease associated to better prognosis [32, 33]. These inconsistencies may be explained by differences in TILs composition across BC subtypes and by changes in TILs composition induced by neoadjuvant antiHER2-containing treatment. A decrease in FOXP3+ TILs has been described in HER2+ tumors achieving pCR, while an increase in FOXP3+ TILs has been described in HER2+ residual disease [34, 35]. Indeed, another study, assessing post-neoadjuvant TILs in 111 HER2+ BC patients treated with chemotherapy+/−trastuzumab, reported that low levels of CD8+ lymphocytes were associated with poor DFS, while low levels of FOXP3+ lymphocytes were associated with better DFS [36].

#### Predictive role of baseline immunity in early HER2+ BC

The ability of TILs to predict trastuzumab benefit appears more controversial (Additional file 1: Table S2). In the FINHER trial [29], 232 patients HER2+ BC were randomized to 9 weeks of trastuzumab in addition to adjuvant chemotherapy. In this study, a significant interaction between TILs and trastuzumab survival benefit was observed, suggesting that trastuzumab might be more efficacious in presence of TILs. The NSABP-31 adjuvant trastuzumab trial randomized HER2+ BC patients to receive doxorubicin-cyclophosphamide followed by paclitaxel+/−trastuzumab. It reported similar results when expression of TIL-associated genes was considered, high expression of TIL-associated genes associated with more benefit from trastuzumab (interaction *p* = 0.03) [28], but did not confirm the interaction between TILs and trastuzumab benefit (*n* = 1581, interaction *p* = 0.556) [30]. Moreover, retrospective analysis of 945 samples from the N9831 trial yield discordant results, as benefit from the addition of trastuzumab was observed in non-LPBC, but not in LPBCs (*n* = 94, interaction *p* = 0.042). However, the number of events in this subgroup was extremely small (*n* = 8). In the same trial, significant benefit from addition of trastuzumab was only observed in immune gene enriched-tumors [24]. The explanation of these discrepancies is currently unknown but might relate to different chemotherapy regimens used in each study, duration of trastuzumab, or concomitant versus sequential trastuzumab administration. Recently, baseline sTILs were assessed in the ShortHER adjuvant trial, which compared 9-weeks versus 1-year trastuzumab in addition to chemotherapy, confirming prognostic value. Moreover, results suggested that low-TILs patients (< 20%) benefited particularly from 1-year trastuzumab over 9-weeks, whereas high-TILs patients experienced an excellent outcome irrespectively of trastuzumab duration (interaction *p* = 0.015) [37].

One important aspect is that TILs might also predict sensitivity to the chemotherapy component. In the Geparsixto trial, 273 HER2+ BC patients received a combination of paclitaxel, anthracycline, trastuzumab, lapatinib +/− carboplatin as neoadjuvant treatment. When 266 HER2+ baseline samples were analyzed [38], not only both sTILs as a continuous variable and LPBC were associated with pCR, but both significantly interacted with the addition of carboplatin (LPBCs showing higher pCR rates when receiving carboplatin). This suggests that TILs might also predict for sensitivity to chemotherapy.

Overall, the current data establishes the clinical validity of pre-existing TILs as a prognostic biomarker. However, more studies are needed to establish the clinical utility of TILs. In early HER2+ BC, several escalation (i.e. adding a second anti-HER2 agent) and de-escalation (i.e. shorter trastuzumab regimens, less-chemotherapy or non-chemotherapy) approaches have been or are being tested. In this context, TILs together with other prognostic clinicopathological variables might allow the construction of prognostic risk models that might help better treat our patients [39].

### Host immunity and other HER2-targeted therapies

Due to their different nature and mechanisms of action, the interaction between immune system and new HER2-targeted treatments might be different from that described with trastuzumab alone (Fig. 1).

#### Pertuzumab

Pertuzumab is a monoclonal antibody directed against the extracellular dimerization domain of HER2 (a different epitope than trastuzumab). Its binding inhibits dimerization of HER2 with other receptors of the HER family. As trastuzumab, pertuzumab can mediate ADCC and simultaneous binding of both antibodies to different HER2 epitopes increases the density of FcγR binding sites on HER2+ cells, possibly enhancing NK-mediated ADCC responses [40]. Consistently, studies on mouse models have reported that combining the two antibodies increases the total number of tumor infiltrating NK-cells and the proportion of them actively engaged in killing tumor cells [41]. In addition, only tumor cells treated with the combination are likely to have a sufficient number of cell-bound antibodies to induce efficient C3 opsonization, required to initiate complement-mediated-cytotoxicity and macrophage-mediated tumor cell killing [42]. However, if this might explain the mechanism of action of pertuzumab or if its improved efficacy in combination with trastuzumab only relies on a more profound pathway inhibition [43] is still unclear.

#### Pertuzumab in the neoadjuvant setting

As pertuzumab only shows significant activity when used in combination with trastuzumab, separating its immune effect in the clinical setting is almost impossible. In the neoadjuvant setting, pertuzumab has been tested in several trials. In the TRYPHAENA trial, testing neoadjuvant pertuzumab and trastuzumab with multi-agent chemotherapy, TILs confirmed their prognostic role. Every 10% increase in baseline TILs was associated with a 25% reduction in DFS hazard, after adjusting for clinicopathological characteristics and pCR. Immune gene-expression signatures were also significantly associated with pCR at multivariate analysis, but not with DFS [44].

The NEOSPHERE trial is a 4-arm study testing neoadjuvant docetaxel in association with trastuzumab, pertuzumab, both or the combination of the two antibodies without chemotherapy. In this trial, baseline TILs as a continuous variable were not significantly associated with pCR, although this might be due to a non-linear effect, as the low TILs group had, as expected, a significantly lower pCR rate. Interestingly, differences were observed across treatment arms. Patients treated with dual anti-HER2 blockade plus docetaxel showed higher rates of pCR, as compared to other treatment arms, in low and intermediate TILs groups, but not in the LPBC group. The impact of immune activation was also explored using gene expression analysis. In the trastuzumab-docetaxel, pertuzumab-docetaxel and in the chemotherapy-free arm, high expression of PDL1, MHC1, and IF-I metagenes associated with lower pCR rates, while high expression of PD1, STAT1, and MHC2 associated with higher pCR rates in multivariable analyses. However, the impact of immune-related metagenes differed across treatment groups. In tumors with high activation of the immune system, the activity of all treatments tested appeared similar (including the chemotherapy-free arm and docetaxel-trastuzumab-pertuzumab arm), while in tumors with low expression of PD1, CTLA4, and MHC1 the use of docetaxel-trastuzumab-pertuzumab was associated with a 2 to 20-fold higher likelihood of pCR as compared to other arms. Indeed, the group in which chemotherapy-trastuzumab performed the least appeared to derive the most benefit from adding pertuzumab [45].

Recently, results from the neoadjuvant PerElisa trial, testing pertuzumab, trastuzumab and letrozole in HR+/HER2+ BC patients selected using Ki67 response after short-course hormonotherapy, were reported. In this trial, baseline TIL levels did not show any impact on pCR [46]. Whether this might be due to the small number of patients, to enrichment in luminal subtypes, to the combination with hormonotherapy or to the absence of chemotherapy remains unclear.

#### Pertuzumab in the metastatic setting

In the metastatic setting, data from the CLEOPATRA trial, testing the addition of pertuzumab to trastuzumab-docetaxel as first-line treatment for HER2+ metastatic BC (mBC), confirmed the positive prognostic role of sTILs. Even if the association between sTILs and progression-free survival (PFS) was not significant, each 10% increase in sTILs significantly associated with longer overall survival (OS). The prognostic effect of TILS appeared to be stronger for OS than for PFS, while no significant interaction with treatment was reported [11]. Two phase III trials assessing addition of pertuzumab to standard treatment in HER2+ mBC, the CLEOPATRA and PHEREXA trials [3, 47], consistently reported a higher magnitude of benefit in terms of OS than of PFS. Enhancement of anti-tumor immune activity by combination of pertuzumab and trastuzumab has been proposed as a possible mechanism for this delayed treatment benefit. Evocatively, a similar benefit in OS with limited benefit in PFS has been described in trials assessing immune checkpoint inhibitors [48, 49]*.*

However, in the CLEOPATRA trial only a small number of patients was pretreated with trastuzumab (10.9%) and most samples analyzed came from primary tumors (93%). In fact, while in untreated BC TILs are associated with a T-effector phenotype, allegedly reflecting an effective antitumour response, immunogenicity is supposed to decrease in the metastatic setting due to activation of immune-evasion mechanisms and to treatment-induced modifications of TME [35]. Consistently, a recent study which assessed sTILs in metastatic samples from 51 HER2+ BCs, mostly pretreated with HER2-targeted agents, did not observe any favorable impact of high sTILs on OS; indeed, a not statistically significant inverse relationship between TILs and prognosis was observed [50].

#### Lapatinib

Lapatinib is a reversible inhibitor of both HER2 and EGFR intracellular tyrosine kinase domains. Due to its intracellular activity, lapatinib might appear to lack the immune activity classically reported with trastuzumab. However, while trastuzumab mediates downregulation and degradation of HER2, lapatinib inhibits phosphorylation of HER2 tyrosine domain, thus preventing ubiquitination. This induces accumulation of HER2 on the cell membrane [51], increasing trastuzumab-dependent ADCC when administered in combination [52]. Lapatinib can also modulate TME. In animal models lapatinib promotes tumor infiltration by CD4 + CD8 + IFN-γ-producing T-cells through a Stat1 dependent pathway. Stat1-deficiency reduces therapeutic activity of lapatinib, suggesting that immune activation can play a role in its antitumor activity [53].

##### Lapatinib in the neoadjuvant setting

Lapatinib has been tested in various clinical settings, either alone or in combination with chemotherapy, trastuzumab or hormonotherapy. Recently, a metanalysis of five neoadjuvant trials reported the impact of TILs in HER2+ BC treated with chemotherapy plus trastuzumab, lapatinib or their combination [54]. Four trials used a combined regimen of anthracyclines and taxanes (CherLOB, GeparQuattro, GeparQuinto and GeparSixto [55–57]), while paclitaxel alone was administered in the NeoALTTO trial [58]. In patients receiving anthracyclines and taxanes, high baseline TILs were significantly associated with pCR, irrespective of the anti-HER2 treatment received (interaction *p* = 0.077). In the NeoALTTO trial, the relationship between TILs and pCR was nonlinear and rates of pCR increased sharply for TIL levels greater than 5% (*p* = 0.01), regardless of treatment group (interaction *p* = 0.519) [58]. However, the relationship between TILs and DFS was linear, regardless of treatment group, and patients with high TILs at baseline had better outcomes independently of whether they achieved pCR. Authors suggested anthracyclines given after surgery might explain the linear relationship with event-free survival.

Immune activation gene signatures were also tested in some of these trials. In the NeoALTTO trial, two T-cell–driven immune signatures significantly associated with pCR. However, this association was only confirmed in multivariable analysis in the combination arm and a significant interaction between these gene signatures and treatment (combination vs single arms) was reported [59]. In the CHERLOB trial, a T-cell gene signature and two immune-related gene signatures significantly correlated with pCR in a multivariate model adjusted by PAM50 [55]. FcγR polymorphisms (FcγRIIa-H131R, FcγRIIIa-V158F) were also tested: only FcγRIIIa V allele carriers showed significant improvement in pCR with dual HER2-blockade (trastuzumab-lapatinib-chemotherapy), and a significant interaction between FcγRIIIa V allele and combination treatment was observed [60]. This might hint to a relevant role for ADCC in determining benefit of combining trastuzumab and lapatinib as compared to single agent, consistently with the significant interaction between immune signatures and combination treatment reported in the NeoALTTO trial [59].

##### Lapatinib in the neoadjuvant setting: Chemo-free combinations

A limited number of trials have tested chemotherapy-free combinations of lapatinib and trastuzumab in metastatic and neoadjuvant setting. The neoadjuvant PAMELA trial treated 151 HER2+ BC patients with trastuzumab-lapatinib (and hormonotherapy if HR-positive) [61]. In this trial, only sTILs at day 15 were significantly associated with pCR at multivariable analysis [9].

##### Lapatinib in the metastatic setting

In the metastatic setting, the CCTG MA.31 trial [62], a phase III trial randomizing 652 HER2+ mBC patients to receive either trastuzumab or lapatinib with taxane, casts an interesting perspective on the role of immunity in modulating activity of HER2-targeted treatment. In this trial, TILs assessed on primary tumor specimens were neither prognostic nor predictive. However, patients with low numbers of CD8+ TILs showed higher risk of progressing when treated with lapatinib compared with trastuzumab (Hazard ratio 2.94; *P* = 0.003) than patients with high CD8+ TILs (Hazard ratio 1.36; *P* = 0.02). The differential effect and a significant interaction was confirmed in a multivariable model [62].

Thus, low CD8+ sTILs in primary tumor predict inferior response to lapatinib vs trastuzumab in the metastatic setting. In fact, the immunogenic state of tumors before and after metastasis is possibly different. As immunogenic tumors present better response to neo/adjuvant treatment, we could speculate that most of the metastatic population will be made up by not immunogenic or immune evasive tumors (probably reflected by low CD8+ sTILs). In this group, trastuzumab can still be expected to function through enhancement of ADCC and priming of antitumor-adaptive T-cell responses. Indeed, previous treatment might modify the immune status of tumors and this should be taken into account in future clinical trials.

#### T-DM1

T-DM1 is an antibody-drug conjugate formed by trastuzumab linked to the cytotoxic agent DM1. After binding HER2, T-DM1 is internalized, degraded in the endosome, releasing DM1. In addition, T-DM1 blocks HER2 signaling pathway and mediates ADCC.

Antitumor immunity might contribute to T-DM1 therapeutic activity. Ansamitocin P3, the precursor of DM1, induces maturation of dendritic cells, facilitates antigen uptake and migration of tumor-resident dendritic cells to tumor-draining lymph nodes, thereby potentiating antitumor immunity [63, 64]. In a HER2+ mouse model, T-DM1 induced infiltration by effector T-cells, which was essential for its therapeutic activity. After T-DM1 treatment (but not with trastuzumab alone), tumors showed a shift towards a T-cell–inflamed phenotype with an increase in γδT-cells and NK-cells and expansion of CD45+. Consistently, the infiltrate showed a strong Th1 immune deviation. In addition, despite the tumor model presented primary resistance to anti–CTLA-4/PD-1 agents, combined use of these agents with T-DM1 resulted in strong antitumor efficacy and in development of immunologic memory [65]. Furthermore, analysis of paired samples from 28 HER2+ BC patients treated with preoperative T-DM1 showed an increase in number and density of tumor-infiltrating T-cells after treatment [65].

#### Neratinib

Neratinib is a pan-HER tyrosine kinase inhibitor. It bonds covalently to a conserved cysteine residue, leading to irreversible inhibition of all four HER receptors, block of downstream pathways and in vitro inhibition of proliferation in tumor cells with trastuzumab resistance [66]. Neratinib recently received approval from FDA and EMA for the extended treatment of early-stage HER2+ BC, based on the phase III ExteNEt trial. This trial reported a small but statistically significant benefit in 5-year DFS for women receiving neratinib for one year after adjuvant trastuzumab versus placebo (90.2% vs 87.7%, *p* = 0.009) [6]. Discordantly from what is observed with pertuzumab and lapatinib, HR+ BC patients appeared to derive greater benefit than HR- patients. It has been suggested that this benefit might rely on intracellular irreversible inhibition of the downstream pathway, potentially limiting crosstalk between the HER2 and endocrine pathways, rendering cells more endocrine-responsive. Despite some evidence suggesting that neratinib can alter HER2 antigen levels, whether this might influence trastuzumab-mediated ADCC remains unknown [67]. Thus, limited available evidence exists regarding the role of immunity in modulating neratinib efficacy.

### Anti-HER2 therapy in breast ductal carcinoma in situ (DCIS)

Anti-HER2 therapy is currently being investigated in DCIS. In a window-of-opportunity trial, patients with HER2-positive DCIS received a single dose of trastuzumab before definitive surgery. No evidence of response was observed. However, trastuzumab augmented NK-cell mediated ADCC, and in one case induced T-cell dependent humoral immunity [68]. A phase III trial of adjuvant trastuzumab in high-risk DCIS (NSABP-43) is currently ongoing to determine if adding trastuzumab to radiotherapy is beneficial in preventing recurrence. Moreover, the effect of tratuzumab on contralateral breast cancer will be evaluated. However, at this time there is no role for routine use of anti-HER2 therapy in DCIS patients.

### Looking deeper: Heterogeneity of HER2+ BC and interaction with immune system

The simplest driver of heterogeneity in HER2+ BC is HR status, differentiating two subgroups with distinct response to HER2-treatment and distinct prognosis. Many immune parameters (i.e. levels of TILs and CD8+ infiltrate) are inversely correlated with HR expression [69], suggesting a reduced immune activity in HR+/HER2+ tumors. In fact, estrogenic signaling interacts with immune activity. Estrogen can regulate the transcription of SerpinB9/proteinase inhibitor 9, a granzyme B inhibitor known to decrease susceptibility of HR + BC cells to NK and CD8+ T-cell cytotoxicity in vitro [70, 71]. Moreover, estrogens might modulate susceptibility of cells to NK-mediated ADCC by upregulating MHC1 transcription [72, 73].

However, HR status does not fully recapitulate heterogeneity in HER2+ BC. In fact, when HER2+ BCs are classified using PAM50 intrinsic subtypes, all subtypes are well represented [61, 74, 75]. The HER2-enriched subtype shows the highest number of mutations [76, 77] and is enriched with high frequency of APOBEC3B-associated mutations [78]. APOBEC-mediated mutagenesis is linked to the acquisition of subclonal mutations [79], genomic instability and potential neoantigens expression. This might explain differences observed in immune infiltrate: in neoadjuvant trials (PAMELA and CHERLOB), HER2-enriched tumors showed the highest levels of TILs as compared to other subtypes, especially Luminal A/B [9, 55]. Luminal and HER2-enriched HER2 + BCs also show genetic differences. The 17q12 chromosomal region, containing genes encoding chemokines and located proximal to the *ERBB2* amplicon, is more frequently coamplified in luminal HER2+ BCs as compared to HER2-enriched HER2+ BCs [80]. Lack of co-amplification, typically observed in HER2-enriched tumors, is associated with higher expression of immune activation and exhaustion-related genes and higher levels of T-cells infiltration [80].

The exceptional sensitivity of HER2-enriched subtype to anti-HER2 treatment, with and without chemotherapy [61, 75], might, at least in part, be due to high immune infiltrate. However, while baseline TILs provide additional independent value to intrinsic subtyping in predicting pCR after neoadjuvant chemotherapy plus HER2-targeted treatment, they have not shown independent predictive value when dual HER2-blockade is used without chemotherapy [9, 46].

### Looking deeper: Interaction with other cancer therapies

HER2-targeted agents are mostly administered in combination with other treatments, such as chemotherapy or endocrine therapy. Even though chemotherapy is often considered immunosuppressive, several cytotoxic agents used in BC, including anthracyclines and cyclophosphamide, induce immunogenic cell death, leading to activation of anti-tumor immune responses. Moreover, cyclophosphamide can reduce the number of circulating T-regulatory cells [81]. In addition, it has been suggested that the synergistic effect of taxanes with trastuzumab might be partly explained by an improvement in NK effectiveness, by up-modulation of NK-activator ligands, and enhancement of trastuzumab-mediated ADCC. Accordingly, NK-cells derived from HER2+ BC patients after taxane-containing therapy expressed higher levels of NKG2D receptor than before [82], suggesting that concomitant administration of taxanes with trastuzumab might maximize the immune effect of the antibody [63, 64].

Less is known about immune-modulating effects of hormonotherapy. In preclinical studies, tamoxifen increased HER2 expression in non HER2-amplified BC cell lines, thus increasing NK cell-mediated ADCC. However, in HR+/HER2-amplified cells, tamoxifen failed to improve NK-cell function, probably because the number of HER2 receptors exceeded the number of FcγRIIIa on NK-cells, already maximizing the potential for NK-cell mediated ADCC [83]. In preclinical trials, the aromatase inhibitor anastrozole has been shown to induce immune activation by inhibiting differentiation of naïve T-cells to T-regulatory, increasing pro-inflammatory and reducing anti-inflammatory cytokines levels [84]. Furthermore, immune activation can be enhanced by the combination of hormonotherapy and CDK4/6 inhibitors. In fact, preclinical data has shown that CdK4/6 inhibitors enhance antitumor immunity by increasing antigen presentation and suppressing proliferation of immunosuppressive T-regulatory cells. Consistently, in patients receiving neoadjuvant palbociclib, an enhanced expression of immune-related signatures was observed [85]. However, efficacy of CdK4/6 inhibitors in HER2+ BC is being tested in clinical trials (NCT02947685, NCT02448420) and these agents are not routinely used in the HER2+ subtype [86].

Most of the clinical information we have regarding the interplay between immune system and hormonotherapy is derived from HR+/HER2- BC [87]. In this setting, gene signatures associated with resistance to tamoxifen, both in the advanced and the adjuvant setting, include immune response genes (*FCGBP, OTUD7B, WFDC2* in the adjuvant, *FCGRT, PSME1, HLA-C, NFATC3* in the advanced setting) [87]. In addition, several inflammation related-genes were identified in gene signatures predictive for poor anti-proliferative response to neoadjuvant aromatase inhibitors [88]. TILs have also been assessed in this context, with discordant results [88, 89].

In a pooled analysis of 3771 patients, baseline TILs as a continuous variable were a predictor of response to neoadjuvant chemotherapy, as evaluated by pCR rates, in all BC subtypes, including HR+/HER2-. Nonetheless, in contrast with HER2+ and TNBC, in HR+/HER2-BC higher TILs associated with shorter OS at multivariate analysis [10]. It has been speculated that the adverse prognostic effect of TILs might be explained by a relative resistance to hormonotherapy. However, extensive data regarding type of hormonotherapy and quantification of residual disease were not available in this study and it cannot be excluded that differences in residual tumor biology might be implicated. For example, in the neoadjuvant chemotherapy GIOB trial, high baseline TILs were associated with a lower rate of Ki67 suppression [89]. Whether a similar effect might be present at least in some subgroups of HR+/HER2+ BC remains unknown.

Finally, the hormonal asset of the patient might play, per se, a modulating role on the immune system. Estrogen signaling has been shown to accelerate progression of various estrogen-insensitive tumor models through mobilization of myeloid-derived suppressor cells and enhancement of their immunosuppressive activity, suggesting that anti-estrogenic agents might boost T-cell-dependent antitumor immunity [90]. Menopausal status of patients might therefore potentially play a modulating role, especially in patients not receiving hormonotherapy.

As interest is growing around the use of chemotherapy-free combinations in HER2+ BC, often containing endocrine agents, an accurate assessment of the interplay between immune system and endocrine therapy in HR+/HER2 + BC is warranted.

### Looking forward: Harnessing the immune system in HER2+ BC

As the contribution of immunity to the activity of HER2-targeted agents has become more apparent, several attempts to exploit it to increase activity or revert resistance to these agents have been made (Table 2).

**Table 2:** Active trials testing immune optimized anti-HER2 treatments for HER2+ BC

Strategy Tested	Study	Phase	Setting	Treatment	N. Patients
Immune-optimized anti-HER2 antibodies	SOPHIA NCT02492711	III	HER2+ mBC progressed on HER2-targeted treatment	Randomized: -Chemotherapy+ Margetuximab -Chemotherapy+ Trastuzumab	530 (active, not recruiting)
Bispecific antibodies	NCT02829372	I	Progressive HER2+ Solid Tumors	GBR1302 (CD3/HER2 bispecific mAb)	60 (recruiting)
Vaccines	NCT03387553	I	During neoadjuvant treatment (HER2+ BC)	HER-2 Pulsed Dendritic cell vaccine	24 (recruiting)
	NCT02061423	I	Post-neoadjuvant residual disease HER-2+ BC	HER-2 Pulsed Dendritic cell vaccine	7 (active, not recruiting)
	NCT02063724	I	Adjuvant (High Risk HER2+ BC)	HER-2 Pulsed Dendritic cell vaccine	15 (active, not recruiting)
	NCT00436254	I	Stage III-IV HER2+ BC or OC	pNGVL3-hICD vaccine (plasmid-based DNA vaccine) + GM-CSF	66 (active, not recruiting)
	NCT01730118	I	Solid tumors with 1–3+ HER2/Neu Expression	Adenoviral Transduced Autologous HER2/Neu Dendritic Cell Vaccine	65 (recruiting)
	NCT01376505	I	Advanced solid tumors	Synthetic peptides of HER-2 comprising B cell epitopes with a Promiscuous T cell epitope of Measles Virus	36 (recruiting)
	NCT01355393	I/II	Stage II-IV HER2+ BC	HER-2/neu peptide vaccine + rintatolimod and/or GM-CSF	50 (active, not recruiting)
	NCT00194714	I/II	Stage IV HLA-A2+ HER2+ BC or OC receiving Trastuzumab	HER2 cytotoxic T-cell peptide-based vaccine	20 (enrolling by invitation)
	NCT01922921	I/II	Stage IV HER2+ BC receiving HER2-targeted mAb	Randomized: -HER2 ICD peptide-based vaccine+polysaccharide-K -HER2 ICD peptide-based vaccine+Placebo	31 (active, not recruiting)
	NCT00343109	II	HER2+ stage IIIB- IV BC receiving trastuzumab	HER-2/neu intracellular domain peptide-based vaccine mixed with GM-CSF	38(active, not recruiting)
	NCT00266110	II	HLA-A0201+ HER2+ mBC	Dendritic cell Vaccine + GM-CSF + trastuzumab + vinorelbine	17(active, not recruiting)
	NCT03384914	II	Adjuvant HER2+ BC	Randomized: -Dendritic Cell (DC1) Vaccine -pUMVC3-IGFBP2-HER2-IGF1R (WOKVAC)	110 (recruiting)
	NCT00640861	NA	Treated Stage II/III MUC1+ HLA-A2+ BC	Randomized: combinations of MUC1/HER-2/Neu Peptide Based Immunotherapeutic Vaccines	45 (active, not recruitng)
	NCT02297698	II	Adjuvant (High Risk HER2+ BC)	Randomized: Trastuzumab/GM-CSF +/− nelipepimut-S	100 (recruiting)
Immune-stimulating agents concomitantly with trastuzumab	NCT03571633	II	Operable HER2+ BC	Randomized: Paclitaxel/trastuzumab +/− Pegfilgrastim	90 (not yet recruiting)
	NCT03112590	I/II	HER2+ BC	IFN-γ + Paclitaxel+Pertuzumab+Trastuzumab	48 (recruiting)
Cellular immunotherapy	NCT02843126	I/II	Recurrent HER2 + BC	Randomized: Trastuzumab +/− NK immunotherapy	30 (recruiting)
	NCT02713984	I/II	Relapsed or refractory HER2+ solid tumors	anti-HER2 CAR-modified T cells	60 (recruiting)

*BC* breast cancer, *CAR* chimeric antigen receptor, *GM-CSF* granulocyte-macrophage colony-stimulating factor, *HLA* human leukocyte antigen, *ICD* intracellular domain, *IFN- γ* interferon gamma, *mAb* monoclonal antibody, *mBC* metastatic breast cancer, *MUC1* mucin1, *N* number, *NA* not available, *NK* natural killer, *OC* ovarian cancer

A first strategy is to optimize ADCC. The interaction between the IgG Fc and the FcγR on an effector cell is the first step leading to immune cell activation and some common single-nucleotide polymorphisms (SNPs) in FcγR genes have been associated with different antibody-binding affinities. Even if reported associations of SNPs with response to trastuzumab are discordant [91–93], attempts have been made to enhance antitumor activity through the design of anti-HER2 antibodies engineered for increased affinity for these SNPs. Margetuximab (MGAH22) is a monoclonal antibody which binds the same epitope of HER2 as trastuzumab, with similar affinities and the same anti-proliferative activity. It carries five aminoacid substitutions in the Fc domain to increase binding to low affinity isoforms of FcγR and reduce binding to CD32B, an inhibitory FccR, resulting in superior engagement of effector cells [94]. Phase I trial testing margetuximab single agent in HER2-overexpressing solid tumors reported meaningful clinical activity [94]. Recently, a press release reported that the phase III trial, comparing the addition to chemotherapy of margetuximab vs trastuzumab in HER2+ mBC patients with progression on prior HER2-targeted treatment, demonstrated a 24% risk reduction in PFS with margetuximab as compared to trastuzumab. However, complete data is still awaited (NCT02492711).

Immune activation can also be enhanced using bispecific antibodies, capable of targeting both HER2 and T-cells, redirecting immune effector cells to the tumor site. Ertumaxomab is a trifunctional bispecific antibody which targets HER2 and CD3. Despite the strong immunologic responses and initial clinical responses observed in HER2+ mBC, the phase II trial was terminated prematurely due to changes in the company’s development plan [95]. Another CD3/HER2 bispecific antibody, GBR 1302, is currently under evaluation in a phase I trial (NCT02829372).

Immunity can also be targeted towards HER2+ cells through HER2 Bi-armed activated T-cells. These T-cells are activated through exposure to murine anti-CD3 monoclonal antibodies and interleukin-2 and then armed with an anti-CD3/anti-HER2 bispecific antibody. This agent was well tolerated in a phase I trial [96] and a phase I/II trial with pembrolizumab is currently ongoing (NCT03272334).

In addition, two phase I/II trials are currently testing autologous HER2 chimeric antigen receptor (CAR)-expressing T-cells in HER2+ mBC and solid tumor patients (NCT02713984, NCT02547961 completed without results). CARs are genetically engineered hybrid T-cell receptors composed by a single-chain variable fragment (scFv) of the B-cell receptor linked to the T-cell receptor CD3ζ transmembrane and intracellular signaling domains plus one or more costimulatory domains, which can induce T-cell mediated cytotoxicity in vitro and regression of tumors in mice models [97]. Moreover, another phase I/II trial is ongoing, testing the combination of trastuzumab and NK immunotherapy in relapsed HER2 + BC (NCT02843126).

With the coming of age of immunotherapy, the use of checkpoint inhibitors to enhance antitumor immunity in HER2+ BC has become an attractive strategy. As preclinical evidence suggests that immune-mediated resistance to trastuzumab can be overcome by combination with checkpoint inhibitors [98], several trials have been testing the association of checkpoint inhibitors and HER2-targeted treatment (Table 3). Preliminary results from the phase I/II PANACEA trial, testing pembrolizumab plus trastuzumab in HER2+ mBC patients who progressed on prior trastuzumab-based therapy, have been presented [12]. In this pretreated population, the combination was active (15.2% overall response rate and 24% clinical benefit rate in the PD-L1+ cohort, no response in the PD-L1- cohort). In the PD-L1+ cohort, baseline sTILs≥5% were significantly associated with objective response (39% vs 5%) and disease control (47% vs 5%).

**Table 3:** Clinical trials testing the association of checkpoint inhibitors and HER2-targeted treatment in HER2 + BC

Study	Phase	Setting	Treatment	N. Patients	Primary outcome evaluated
NCT02605915 Cohort 2A	Ib	Neoadjuvant HER2+ BC	Atezolizumab/Trastuzumab/Pertuzumab followed by docetaxel + carboplatin + trastuzumab + pertuzumab	98 entire trial (recruiting)	Safety
NCT02605915 Cohort 2B	Ib	Neoadjuvant HER2+ BC	Atezolizumab + T-DM1 followed by docetaxel + carboplatin + trastuzumab + pertuzumab		
NCT02605915 Cohort 1A	Ib	locally advanced or mHER2+ BC	Atezolizumab/Trastuzumab/Pertuzumab		
NCT02605915 Cohort 1B-C-D	Ib	locally advanced or mHER2+ BC	Atezolizumab + T-DM1		
NCT02605915 Cohort 1F	Ib	locally advanced or mHER2 + BC	Atezolizumab/Trastuzumab/Pertuzumab/ Docetaxel		
NCT03032107	I	mHER2+ BC	T-DM1 + Pembrolizumab	27 (recruiting)	Safety
NCT02649686	Ib	mHER2+ BC	Trastuzumab + Durvalumab	15 (active, not recruiting)	Safety
NCT03272334	I/II	mHER2 + BC	Pembrolizumab + Anti-CD3 x Anti-HER2 Armed Activated T Cells	33 (recruiting)	Safety
NCT02129556 (PANACEA)	Ib/II	Unresectable or mHER2+ BC	Pembrolizumab + Trastuzumab	58 (active, not recruiting)	Phase I: Safety Phase II: Response by RECIST
NCT03417544	II	mHER2+ BC with brain mts	Atezolizumab + trastuzumab + pertuzumab	33 (recruiting)	Overall Response Rate by RANO-BM criteria
NCT03125928	II	Unresectable or mHER2+ BC	Atezolizumab + paclitaxel + trastuzumab + pertuzumab	50 (recruiting)	Safety and Response by RECIST
NCT03414658	II	mHER2 + BC progressed to prior trastuzumab and pertuzumab	Randomized: -Trastuzumab/Vinorelbine -Trastuzumab/Vinorelbine+Avelumab - Trastuzumab/Vinorelbine+Avelumab +Utomilumab	100 (recruiting)	PFS
NCT03199885	III	mHER2 + BC	Randomized: -Paclitaxel/Trastuzumab/Pertuzumab + Pembrolizumab -Paclitaxel/Trastuzumab/Pertuzumab	480 (not yet recruiting)	PFS

*BC* breast cancer, *mBC* metastatic breast cancer, *N* number, *PFS* progression-free survival, *RANO-BM* response assessment in neuro-oncology – brain metastases

On the other hand, the KATE2 phase II trial failed to demonstrate an overall PFS benefit from adding atezolizumab to T-DM1 in HER2+ mBC. However, a PFS benefit for the combination was present in PD-L1+ and high CD8+ TILs tumors, although the magnitude of benefit was uncertain given the limited number of patients [99].

Several studies testing immune checkpoint inhibitors in combination with HER2-targeted therapies are currently ongoing (Table 3). These will help us understand better the interaction between immune system and HER2-targeted agents and define successful combinations for future clinical trials.

## Conclusions

The role of immunity in cancer treatment has recently moved into the spotlight, as mechanisms related to immune surveillance, immune equilibrium, and immune escape have progressively been elucidated in several solid tumors and new drugs have entered clinical practice. Recent evidence from early phase trials supports the therapeutic role of immunity in HER2 + BC and more data from ongoing trials will be available in the next few years.

In HER2+ BC, the interplay between immune system and tumor is complex and dynamic, involving the interaction with different HER2-targeted treatments, chemotherapy, hormonotherapy and the modulating action of HR status and tumor biology. A deeper understanding of these mechanisms might help optimize treatment personalization in HER2+ BC and design biologically meaningful trials that will eventually change the way we treat patients with HER2+ disease.

## Additional file

Additional file 1: **Table S1.** Summary of prognostic/predictive value of tumor infiltrating lymphocytes (TILs) in HER2+ breast cancer across prospective interventional clinical trials. **Table S2.** Summary of prognostic/predictive value of expression of immune genes and immune gene signatures in HER2+ breast cancer across prospective interventional clinical trials. **Table S3.** Peptide-based vaccine strategies targeting HER2 for the treatment of invasive HER2+ BC (early and advanced setting). (DOCX 48 kb)
