# Supplementary material for: Interaction of host immunity with HER2-targeted treatment and tumor heterogeneity in HER2-positive breast cancer
Source: J Immunother Cancer. 2019 Mar 29;7:90. doi: 10.1186/s40425-019-0548-6 (PMC6439986; doi:10.1186/s40425-019-0548-6)
Supplement: Supplementary file 1 — Table S1. Summary of prognostic/predictive value of tumor infiltrating lymphocytes (TILs) in HER2+ breast cancer across prospective interventional clinical trials. Table S2. Summary of prognostic/predictive value of expression of immune genes and immune gene signatures in HER2+ breast cancer across prospective interventional clinical trials. Table S3. Peptide-based vaccine strategies targeting HER2 for the treatment of invasive HER2+ BC (early and advanced setting). (DOCX 48 kb) [file 40425_2019_548_MOESM1_ESM.docx]

**Supplementary Table 1: Summary of prognostic/predictive value of tumor infiltrating lymphocytes (TILs) in HER2+ breast cancer across prospective interventional clinical trials**

| **Trial** | **Author** | **Time** | **No. of patients** | **Treatment** | **Cut-off value** | **Outcome** | **Association with outcome** | **Notes** |
| --- | --- | --- | --- | --- | --- | --- | --- | --- |
| **CherLOB[54]** | Dieci *et al.* | Neoadjuvant setting | 105 | P + H/L/HL  → FEC + H/L/HL | 1% INC | pCR | Yes | Association only at univariate analysis |
|  |  |  |  |  |  | EFS | Yes |  |
| **GeparQuattro[57]**  **and GeparQuinto[57]** | Heppner *et al.* | Neoadjuvant setting | 178 | AC-H → D-H +/-X | 10% INC  And  LPBC ≥ 60% | pCR | Yes | No significant association with DFS |
|  |  |  | 162 | AC-H → D-H |  |  | Yes |  |
|  |  |  | 158 | AC-L → D-L |  |  | No |  |
| **GeparSixto[55]** | Denkert *et al.* | Neoadjuvant setting | 266 | PM-HL +/- Cb | LPBC≥ 60% | pCR | Yes | Interaction with carboplatin benefit |
| **NeoALTTO[58]** | Salgado *et al.* | Neoadjuvant setting | 387 | P+ H/L/HL | >5%, | pCR | Yes | No interaction with treatment |
|  |  |  |  |  | 1% INC | EFS | Yes |  |
| **NeoSphere[44]** | Bianchini *et al.* | Neoadjuvant setting | 243 | DH  DPrtz  DHPrtz  HPrtz | 1% INC | pCR | No |  |
| **PAMELA[9]** | Nuciforo *et al.* | Neoadjuvant setting | 134 | H+L | 1% INC baseline | pCR | Yes | Only at univariate |
|  |  |  |  |  | 1% INC at day15 | pCR | Yes |  |
| **Tryphaena[101]** | Ignatiadis *et al.* | Neoadjuvant setting | 213 | FEC → DHPrtz  FEC+HPrtz→DHPrtz  TC + HPrtz | 10% INC | pCR | No |  |
|  |  |  |  |  |  | EFS | Yes |  |
| **FINHER [28]** | Loi *et al.* | Adjuvant setting | 209 | D/V-FEC +/- H | 10% INC | DDFS | Yes  (in pts receiving H) | Interaction with treatment |
| **N9831 [27]** | Perez *et al.* | Adjuvant setting | 945 (489 not receiving H) | AC → P +/-H | 10% INC and  LPBC ≥ 60% | RFS | Yes  (in pts receiving not H) | Interaction with treatment |
| **NSABP-31[30]** | Kim *et al*. | Adjuvant setting | 1581 | AC → P +/-H | 10% INC and  LPBC ≥ 50% | DFS | Yes | No interaction with treatment |
| **ShortHER[37]** | Dieci *et al*. | Adjuvant setting | 866 | AC/FEC → D + H (9 wks vs 1 yr) | 10% INC and  ≥ 20% | DDFS | Yes | Interaction with treatment |
| **CLEOPATRA**  **[11]** | Luen *et al.* | Metastatic  First line | 678 | D + H/H+Ptz | 10% INC | PFS | No | Not predictive for pertuzumab benefit |
|  |  |  |  |  |  | OS | Yes |  |
| **MA.31**  **[62]** | Liu *et al.* | Metastatic  First line | 614 | T + H/L | ≥5% | PFS | No |  |
|  |  |  |  |  |  | OS |  |  |

Abbreviations: 10% INC, 10% increment; LPBC, lymphocyte-predominant breast cancer; AC, anthracycline-cyclophosphamide; Cb, carboplatin; CMF, cyclophosphamide-methotrexate-fluorouracil; Cyclo, cyclophosphamide; D, docetaxel; DFS, disease-free survival; DDFS, distant disease-free survival; EC, epirubicin-cyclophosphamide; EFS, event-free survival; FEC, fluorouracil-epirubicin-cyclophosphamide; H, trastuzumab; L, lapatinib; MFS, metastasis-free survival; OS, overall survival; P, paclitaxel; pCR, pathologic complete response; PFS, progression-free survival; Pl, placebo; PM, weekly paclitaxel + non pegylated liposomal doxorubicin; Prtz, pertuzumab; RFS, recurrence-free survival; T, taxanes; TC, taxanes-cyclophosphamide; TIL, tumor infiltrating lymphocytes; V, vinorelbine; wks, weeks; X, capecitabine; yr, year.

**Supplementary Table 2: Summary of prognostic/predictive value of expression of immune genes and immune gene signatures in HER2+ breast cancer across prospective interventional clinical trials**

| **Trial** | **Author** | **Time** | **No. of patients** | **Treatment** | **Gene Signatures Tested** | **Outcome** | **Association with outcome** |
| --- | --- | --- | --- | --- | --- | --- | --- |
| **CALGB 40601[100]** | Carey *et al.* | Neoadjuvant setting | 265 | P-H  P-L  P-HL | Immune gene signatures[97] | pCR | IgG signature independently associated with pCR at multivariate analysis |
| **CherLOB[54]** | Dieci *et al.* | Neoadjuvant setting | 86 | P + H/L/HL  → FEC + H/L/HL | Immune gene signatures[55] | pCR | 3 out of 4 signatures maintained association with pCR after correction for PAM50 |
| **GeparSixto[55]** | Denkert *et al.* | Neoadjuvant setting | 226 | PM-HL +/- Cb | mRNA expression of immunologic genes | pCR | All 12 immune mRNA markers were associated with pCR (10/12 at multivariate analysis) |
| **NeoALTTO[59]** | Fumagalli *et al.* | Neoadjuvant setting | 254 | P+ H/L/HL | Immune gene signatures[60] | pCR | two T-cell immune signatures were associated with pCR (only confirmed at multivariate analysis in P-HL arm) |
| **NOAH[44]** | Bianchini *et al.* | Neoadjuvant setting | 51 | AP → P → CMF | Four immune metagenes[44] | pCR | Not associated with pCR |
|  |  |  | 63 | AP-H →P-H →CMF-H →H |  | pCR | 3/4 associated with pCR |
| **NeoSphere[44]** | Bianchini *et al.* | Neoadjuvant setting | 337 | DH  DPrtz  DHPrtz  HPrtz | Immune genes and metagenes[44] | pCR | 5 associated with pCR at multivariate analysis* |
| **Tryphaena[101]** | Ignatiadis *et al.* | Neoadjuvant setting | 173 | FEC → DHPrtz  FEC+HPrtz→DHPrtz  TC + HPrtz | Immune signatures and genes | pCR | 2 signatures and 4 genes associated with pCR at multivariate analysis |
|  |  |  |  |  |  | EFS | Not associated with EFS at multivariate analysis |
| **FINHER [Sup1]** | Schmidt *et al.* | Adjuvant setting | 199 | D-FEC +/- H | CXCL13, FOXP3 and CD4 RNA expression | DDFS | No |
| **N9831 [24]** | Perez *et al.* | Adjuvant setting | 849 | AC → P+/-H → H | Immune-response enriched signature | RFS | Yes* |
|  |  |  | 433 | AC → P |  |  | No |

*In the NeoSphere trial, interaction with treatment (different results in the DHPrtz arm) was reported. In the N9831 study, interaction with having or not received trastuzumab was reported.

A, doxorubicin; AC; anthracycline-cyclophosphamide; C, carboplatin; CMF, cyclophosphamide-methotrexate-fluorouracil; D, docetaxel; EFS, event-free survival; FEC, fluorouracil-epirubicin-cyclophosphamide; H, trastuzumab; L, lapatinib; P, paclitaxel; pCR, pathologic complete response; PM, weekly paclitaxel + non pegylated liposomal doxorubicin; Prtz, pertuzumab; V, vinorelbine.

**Supplementary Table 3: Peptide-based vaccine strategies targeting HER2 for the treatment of invasive HER2+ BC (early and advanced setting)**

| **Agent** | **Description** | **Type** | **Setting** | **Phase I** | **Phase II** | **Phase III** |
| --- | --- | --- | --- | --- | --- | --- |
| E75 | nine amino acids from the extracellular domain of HER2 | Peptide-based vaccine | Metastatic | In combination with GM-CSF:  Completed. Immunity confirmed [Sup2] |  |  |
|  |  |  | Adjuvant | Phase I/II in combination with GM-CSF: non statistically significant benefit (5-year DFS rate 89.7% for vaccinated vs 80.2% for control patients; p=0.08) [Sup3] | In combination with GM-CSF and trastuzumab in high-risk HER2+ BC: ongoing (NCT02297698) | * |
| GP2 | nine amino acid peptide from the trans-membrane portion of HER2 | Peptide-based vaccine | Adjuvant | In combination with GM-CSF: safety and immunogenicity confirmed [Sup4] | Randomized:  GP2+GM-CSF vs GM-CSF alone in HLA-A2–positive patients with high-risk BC: no overall benefit  In the HER2+ subgroup per-treatment DFS was 100 % in the vaccine group vs 89 % in the control group (*p* = 0.08) [Sup5] |  |
|  |  |  |  | In combination with GM-CSF and trastuzumab: safety and immunogenicity confirmed [Sup6] |  |  |
| AE37 | Li-Key/HER2 hybrid (couples HLA I HER2 peptide with MHC class II epitopes) | Peptide-based vaccine | Metastatic |  |  |  |
|  |  |  | Adjuvant | In combination with GM-CSF: immunogenicity and safety demonstrated [Sup7] | Randomized:  AE37 + GM-CSF vs GM-CSF alone   in high-risk BC (any degree HER2 expression): no overall benefit  Possibly a non significant benefit in the triple-negative cohort [Sup8] |  |
| dHER2 | Recombinant protein: consisting extracellular domain + a portion of the intracellular domain of HER2 combined | Protein-based vaccine | Metastatic | In combination with AS15: immunity confirmed [Sup9] |  |  |
|  |  |  | Stage II–III HER2+ BC trastuzumab naive | Phase I/II in combination with AS15: immunity confirmed (10/40 patients achieved prolonged stable disease. [Sup10] | |  |

*****The adjuvant phase III PRESENT trial only enrolled patients with low expression of HER2 BC and was closed due to futility at preplanned analysis

**References**

Sup1. Schmidt M, Weyer-Elberich V, Hengstler JG, Heimes A-S, Almstedt K, Gerhold-Ay A, et al. Prognostic impact of CD4-positive T cell subsets in early breast cancer: a study based on the FinHer trial patient population. Breast Cancer Res. 2018;20(1):15.

Sup2. JL Murray, ME Gillogly, D Przepiorka. Toxicity, immunogenicity, and induction of E75‐specific tumor‐lytic CTLs by HER‐2 peptide E75 (369–377) combined with granulocyte macrophage colony‐stimulating factor in HLA‐A2+ patients with metastatic breast and ovarian cancer. Clin Cancer Res. 2002;8(11):3407-18.

Sup3. Peoples GE, Gurney JM, Hueman MT, Woll MM, et al. Clinical trial results of a HER2/neu (E75) vaccine to prevent recurrence in high-risk breast cancer patients. J Clin Oncol. 2005; 23:7536–7545

Sup4. Carmichael MG, Benavides LC, Holmes JP, Gates JD, Mittendorf EA, Ponniah S, Peoples GE. Results of the first phase 1 clinical trial of the HER-2/neu peptide (GP2) vaccine in disease-free breast cancer patients: United States Military Cancer Institute Clinical Trials Group Study I-04. Cancer. 2010;116(2):292-301.

Sup5. Mittendorf EA, Ardavanis A, Litton JK, Shumway NM, Hale DF, Murray JL, et al. Primary analysis of a prospective, randomized, single-blinded phase II trial evaluating the HER2 peptide GP2 vaccine in breast cancer patients to prevent recurrence. Oncotarget. 2016;7(40):66192-66201.

Sup6. Clifton GT, Litton JK, Arrington K, Ponniah S, Ibrahim NK, Gall V, et al. Results of a Phase Ib Trial of Combination Immunotherapy with a CD8+ T Cell Eliciting Vaccine and Trastuzumab in Breast Cancer Patients. Ann Surg Oncol. 2017;24(8):2161-2167.

Sup7. Holmes JP, Benavides LC, Gates JD, Carmichael MG, Hueman MT, Mittendorf EA, et al. Results of the first phase I clinical trial of the novel II-key hybrid preventive HER-2/neu peptide (AE37) vaccine. J Clin Oncol. 2008;26(20):3426-33.

Sup8. Mittendorf EA, Ardavanis A2 Symanowski J, Murray JL, Shumway NM, Litton JK, et al. Primary analysis of a prospective, randomized, single-blinded phase II trial evaluating the HER2 peptide AE37 vaccine in breast cancer patients to prevent recurrence. Ann Oncol. 2016;27(7):1241-8..

Sup9. Hamilton E, Blackwell K, Hobeika AC, Clay TM, Broadwater G, Ren XR, et al. Phase 1 clinical trial of HER2-specific immunotherapy with concomitant HER2 kinase inhibition. J Transl Med. 2012;10:28.

Sup10. Limentani SA, Campone M, Dorval T, Curigliano G, de Boer R, Vogel C, White S, et al. A non-randomized dose-escalation Phase I trial of a protein-based immunotherapeutic for the treatment of breast cancer patients with HER2-overexpressing tumors. Breast Cancer Res Treat. 2016;156(2):319-30.
